# Progress in Surgery

**Published:** 1898-09-03

**Authors:** 


### Progress in Surgery.

### RENAL SURGERY.

The principle of exposing the kidney by separation rather than division of muscular fibres is advocated by Mayo Robson, although it has been practised by others also. This incision is from the anterior superior spine to the top of the last rib in the direction of the fibres of the external oblique, which are separated. The internal oblique fibres are separated in a line between the ninth costal cartilage and the posterior superior spine of the ilium, and the transversalis fibres are split at the same time. Robson gives several examples, and claims the following advantages: (1) No division of muscle and no weakening; (2) no division of vessels or nerves; (3) the operation is done with the patient on the back; (4) the danger is diminished and convalescence shortened; (5) the operation can be continued so as to explore the whole of the ureter down to the bladder.[1]

The application of skiagraphy to the diagnosis of renal calculus has proved fruitful in many cases. Mr. C. A. Morton describes a case in which the symptoms of calculus became quiescent, yet the X-rays detected a calculus and led to its successful removal.[2]

Fixation of moveable kidneys is not always successful, and rarely so unless the operation be thorough. Senn advocates a method of free exposure and scarification of the kidney, its support by a sling of iodoform gauze, and packing of the wound with gauze. Simon's vertical lumbar incision is employed, and the fatty capsule is removed from the back of the kidney. The kidney is well scarified, the lower end brought into the wound and kept there by a sling composed of four layers of gauze. Then the wound is packed with gauze, the ends of the sling being tied over the packing. No sutures are used, and the wound is allowed to granulate, the packing being removed towards the end of the week.[3]

As shown by Mr. Henry Morris in the lectures to which we shall presently refer, exploratory operations on the kidney are comparatively safe and clearly indicated. Sometimes, indeed, it is advisable to remove a piece of renal tissue for microscopic examination. This has been repeatedly practised by M. Bloch.[4] Renal tumours have in his hands thus shown to be forms of nephritis, glomerular, interstitial, &c. Moreover, renal hæmaturia may occur without discoverable lesion of the kidneys. Dr. Harris (Chicago) details 16 such cases. The hæmaturia is almost invariably unilateral, so that a local lesion may be suspected, and nephrotomy usually leads to a cessation of the symptoms.[5]

Gonorrhœal pyelitis may exist with few symptoms, and even when there is little affection of the bladder.[6] The chief indication is the presence in the urine of epithelium from the renal pelvis, with blood and pus microscopically. Its early discovery and treatment may lead to cure. Attention is directed to the possible early occurrence of this trouble without at first any marked symptoms.

The recent Hunterian lectures at the College of Surgeons, by Mr. Henry Morris, called forth a leader in the *Lancet*, which states that the first real operation on the kidney was performed in 1869 by Gustav Simon. The early operation for renal calculus was first performed by Morris in 1880. He has removed calculi in 34 cases with only one death. The surgery of the ureter is novel and revolutionary; ureteral anastomosis, for instance, now substitutes nephrectomy. It may be interesting to give Mr. Morris's results and tables:[7]

| | | | |
|---|---|---|---|
| Nephrolithotomy | ... 34 | Cases | 1 death. |
| Nephrotomy for stone | ... 44 | ,, | 11 deaths within a short time. |
| Nephrectomy for stone | ... 18 | ,, | 5 deaths. |
| Exploratory operations | ... 42 | ,, | 2 deaths. |
| Moveable kidney | ... 57 | ,, | No deaths. |
| Hydro- and pyo-nephros's | 20 | Cases | 3 deaths. |
| Tuberculous disease | ... 28 | ,, | 9 deaths within a comparatively short time. |
| Nephrectomy for fistula, &c. | 5 | ,, | 2 deaths. |
| For tumours of kidney | ... 15 | ,, | 6 deaths. |
| Injury to kidney | ... 4 | ,, | No deaths. |
| Calculus anuria | ... 49 | ,, | 29 deaths. |

It must be remembered that many of these conditions are fatal without surgical treatment.

**Catheterisation of the Ureters.**—Dr. Howard Kelly has applied his method of direct examination of the bladder and ureters to the male sex, and has demonstrated its utility. He uses a long speculum blackened on the inside. The patient is first placed in the knee-breast position, then a speculum is passed into the rectum to distend it with air, and cause the base of the bladder to descend within easy reach of inspection. The speculum is then passed through the retroverted penis, and on removal of the obturator the bladder distends with air, and the trigone and posterior wall can be examined. By the introduction of a mirror with a long handle the apex of the bladder can be examined.[8]

This methods gives facilities both for catheterisation of the ureter and renal pelvis, and for the local treatment of morbid conditions of the bladder.

[1] Lancet, May 14, 1898. [2] Lancet, June 4, 1898. [3] Amer. Jour. Med. Sci., May, 1898. [4] La Semaine Médicale, Feb. 26, 1898. [5] Med. Chron., April, 1898. [6] Med. Rec., June 25, 1898. [7] Lancet, May 14, 1898. [8] Annals of Surgery, April, 1898.

